# TiO_2_-Modified Magnetic Nanoparticles (Fe_3_O_4_) with Antibacterial Properties

**DOI:** 10.3390/ma15051863

**Published:** 2022-03-02

**Authors:** Agnieszka Wojciechowska, Agata Markowska-Szczupak, Zofia Lendzion-Bieluń

**Affiliations:** Faculty of Chemical Technology and Engineering, West Pomeranian University of Technology in Szczecin, Aleja Piastów 42, 71-065 Szczecin, Poland; agnieszka-wojciechowska@zut.edu.pl (A.W.); agata.markowska@zut.edu.pl (A.M.-S.)

**Keywords:** iron oxide, titanium dioxide, antibacterial properties

## Abstract

This paper presents the synthesis and characteristics of Fe_3_O_4_/C/TiO_2_ hybrid magnetic nanomaterials with antibacterial properties. The materials used were obtained using a microwave-assisted two-stage precipitation method. In the first stage, magnetite nanoparticles (Fe_3_O_4_) were prepared with the precipitation method, during which an additional glucose layer was placed on them. Next, the surface of Fe_3_O_4_ nanoparticles was covered by TiO_2_. It was observed that the addition of carbon and titanium dioxide caused a decrease in the average size of magnetite crystallites from 15.6 to 9.2 nm. Materials with varying contents of anatase phase were obtained. They were characterized in terms of phase composition, crystallite size, specific surface area, surface charge and the kinds of function groups on the surface. The results show a successful method of synthesizing hybrid magnetic nanoparticles, stable in a solution, with antibacterial properties under direct solar light irradiation. Compared to classical materials based on TiO_2_ and used for water disinfection, the obtained photocatalytic nanomaterials have magnetic properties. Owing to this fact, they can be easily removed from water once their activity under direct irradiance in a given process has completed.

## 1. Introduction

Over recent years, the incidence and mortality rates of infectious diseases caused by airborne pathogenic bacteria have been on the increase. Such infections are the cause of a minimum of 4 million deaths annually [1]. Another pressing problem the world has experienced recently is antibiotic resistant bacteria (ARB). Drug-resistant microbes occur in surface waters, domestic, hospital sewage and waste from animal farms and pharmaceutical production plants that are biologically treated. Antibiotic-resistant strains of bacteria have higher resistance to commonly used disinfectants, including ozone and chlorine compounds. 

In light of the SARS CoV-2 pandemic, the danger and problems posed by the uncontrolled transmission of pathogens seem obvious [2]. Therefore, experts from many various fields seek methods to synthesize novel antimicrobial medication that would be a successful tool in the fight against pathogenic microbes. Some nanomaterials, including metal oxides, with antimicrobial properties have already been developed owing to interdisciplinary research. Their high specific surface area maximizes interaction with microbiological membrane. Metal oxides are characterized by a low toxicity, high stability and higher selectivity compared to other, particularly organic, materials [3]. The main mechanism of their microbial inhibition is to trigger oxidative stress by boosting the amount of reactive oxygen species (ROS) [4]. 

One of the aims behind the search for novel treatment methods for diseases caused by pathogenic microbes is to improve drug transport by reducing the concentration or by controlled drug distribution [5]. 

Magnetite nanoparticles can potentially be used as antibacterial agents owing to their high biocompatibility, nontoxicity, large specific surface area and strong magnetic properties [6]. However, magnetite nanoparticles are prone to agglomeration as the particle size gets smaller, which inhibits their antimicrobial activity. Additionally, for biomedical applications, the surface of magnetite nanoparticles needs to be modified to more easily obtain a water suspension with high stability and to resist the effect of protein and salt in the physiological environment [7]. 

The modification of magnetite nanoparticles can be conducted with materials that can produce carbon coating, e.g., with sugars [8]. The aim of this process is to deliver functional groups to the surface of magnetic nanoparticles. Functional groups can improve the stability of newly formed nanostructures and can help to bind them to nanoparticles that form the new hybrid material, which can have photocatalytic and antibacterial properties. Moreover, the new intermediate layer of carbon between magnetite and TiO_2_ nanoparticles improves the photoactivity of the material under visible light conditions [9]. 

For the above reasons, it is thought that developing a synthesis of hybrid nanoparticles, based on magnetite with high stability, that can be well dispersed in an aquatic environment can be a significant step forward in the search for new materials with antimicrobial properties. 

Hybrid magnetic nanoparticles connecting the unique properties of their components are widely used in wastewater treatment, as drug delivery agents, and to remove organic impurities in photocatalytic degradation processes [10,11].

This paper presents the synthesis and characteristics of magnetic nanomaterials based on iron (II and III) oxide nanoparticles covered by a layer of surfactant and titanium dioxide. The antibacterial properties of the new material against model Gram-negative and Gram-positive bacteria were also tested.

## 2. Materials and Methods

### 2.1. Synthesis of Fe_3_O_4_/C/TiO_2_

The material used in our experiments was obtained with a two-stage method. The first stage was the synthesis of pure magnetite using the microwave-assisted precipitation method. The salt: iron (III) chloride and iron (II) chloride were used as the source of iron. Ammonia water (25% solution) was used as the precipitating agent. In the second stage, the microwave-assisted impregnation method was used to cover the surface of magnetite nanoparticles with a layer of carbon and titanium dioxide.

Both processes were conducted in a microwave MAGNUM II reactor manufactured by ERTEC (Wroclaw, Poland). A glucose to magnetite phase molar ratio of 0.2:1 was used as the precursor of the carbon layer. Titanium tetrabutoxide was used as the precursor of titanium. A detailed description of the method of obtaining Fe_3_O_4_/C/TiO_2_ materials was presented elsewhere [10]. The starting material, i.e., pure magnetite and magnetite modified with titanium dioxide in different magnetite to titanium (IV) dioxide molar ratios of 1:1, 1:3 and 1:10 and a constant content of carbon layer were examined.

### 2.2. Characteristics of Obtained Materials

The X-ray powder diffraction method was used to determine the phase composition of obtained materials with an Empyrean (Malvern Panalytical, Malvern, UK) diffractometer in a Bragg–Brentano geometry, equipped with a monochromatic radiation source Cu Kα (λα1 = 0.154056 nm, λα2 = 0.154439 nm). The PANalytical High Score Plus v.3.0e software (Malvern Panalytical) with ICDD PDF4+ database was used for data analysis. A full-pattern fit based on the Rietveld refinement was used to calculate the weight fractions of the identified crystallographic phases. The Scherrer equation was used to determine the size of magnetite and anatase crystallites [11,12,13]:

$$D=\frac{k\lambda }{\beta cos\theta }$$

where *D* is the crystallite size, *k* is the dimensionless shape factor, λ is the X-ray wavelength, β is the line broadening at half maximum intensity, and θ is the Bragg angle.

Specific surface area, total pore volume and pore distribution were determined based on low-temperature nitrogen sorption using Quantachrome Instruments (Boynton Beach, FL, USA). Before measurement, samples were dried and degassed at 140 °C for 12 h under high vacuum. The surface area was determined using multipoint Brunauer–Emmet–Teller (BET) analysis of adsorption isotherms.

Fourier-transform infrared spectroscopy (FT-IR) was used to identify functional groups on the surface of obtained materials. FT-IR spectra were measured in the range of 4000–400 cm^−1^ with a Thermo Scientific Nicolet 380 instrument (Madison, WI, USA).

The isoelectric point and zeta potential of Fe_3_O_4_ and Fe_3_O_4_/C/TiO_2_ dispersions in ultrapure water were determined using a Zetasizer Nano-ZS (Malvern Instruments Ltd., Malvern Panalytical, Malvern, UK) equipped with an MPT-2 multi-purpose titrator and a degasser. The pH was adjusted using HCl and NaOH solutions.

In order to explore the antibacterial activities of synthesized Fe_3_O_4_/C/TiO_2_ materials, the Gram-negative bacterium *Escherichia coli* K12 (ATCC 25922, Manassas, VA, USA) and the Gram-positive bacterium *Staphylococcus epidermidis* (ATCC 4946, Manassas, VA, USA) were used. The bacterial isolates were cultured overnight in nutrient broth (BioMaxima S.A., Lublin, Poland) for *E. coli* and Brain Heart Infusion Broth BHI (BioMaxima S.A., Lublin, Poland) for *S. epidermidis* at 37 °C and 120 RPM in a rotary shaker. After centrifugation (4000 RPM, for 10 min), the bacterial cultures were diluted with saline (0.85% NaCl) or Phosphate-buffered saline (PBS, Chemland, Stargard, Poland), sterile buffers suitable for bacteria species. The final concentration of working bacterial dilution was 0.5 in McFerland standard (bioMérieux, Warsaw, Poland), approximately 1.8 × 10^8^ CFU/mL. The tests were conducted in a 100 mL sterilized glass container containing bacterial suspension (1 mL working solution containing the bacteria) and 0.1 g/dm^3^ of the Fe_3_O_4_/C/TiO_2_ rotating at 200 RPM. The suspension was irradiated with a lamp emitting artificial solar light UV-Vis (ULTRA-VITALUX 230V E27/ES, OSRAM 300W) or kept in the dark (control experiment) at 37 °C. The distance between the solution and the light source was fixed at ca. 15 cm. Serial dilutions (10^−1^ to 10^−6^) were prepared in a saline solution (0.85%) after 1, 2 and 3 h. Then, 0.5 mL of the solutions was cultured on an agar plate and placed on Plate Count Agar (PCA agar, BTL, Lodz, Poland) for *E. coli* or Brain Heart Agar for *S. epidermidis*. Agar plates were incubated at 37 °C overnight, and then the bacterial colonies were counted to calculate the colony-forming unit/mL (CFU/mL). All cultures were repeated three times. Control experiments for bacterial suspensions were also conducted.

## 3. Results and Discussion

**Figure 1:**
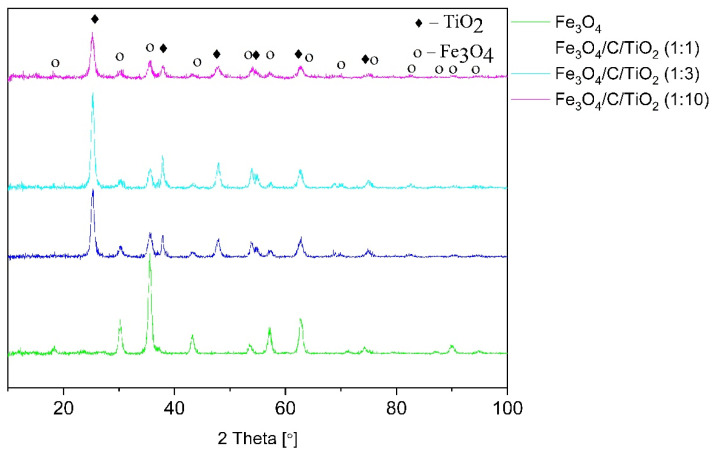
XRD patterns of pure magnetite and titanium dioxide-modified magnetite in various molar ratios Fe_3_O_4_/C: TiO_2_ and constant glucose content.

Figure 1 shows diffractograms of pure magnetite, titanium dioxide-modified magnetite in different molar ratios, and magnetite covered with a constant amount of glucose compared to titanium dioxide. The diffractograms of pure magnetite show characteristics reflexes at the angles of 18.32, 30.21, 35.57, 43.24, 53.64, 57.16, 62.71, 71.15, 74.20, 82.13, 87.00, 89.91 and 94.76, which correspond to the crystallographic planes of [111], [220], [311], [400], [422], [511], [440], [620], [533], [711], [642], [731] and [800] of the magnetite phase (ICDD: 04-006-6550).

This pattern corresponds to the cubic structure of Fe_3_O_4_ (Fd-3m space group), with the lattice constants of a = b = c= 8.374 Å. Materials modified with titanium dioxide had characteristic reflexes at the angles of 25.26, 37.84, 47.89, 54.89, 62.93 and 75.41, which correlated with the crystallographic planes of [101], [103], [200], [105], [204] and [215], typical of the anatase phase with tetragonal structure with the lattice constants of a = 3.79 Å, b = 3.79 Å, and c = 9.41 Å (ICDD:01-075-2550). The material with the magnetite to titanium dioxide molar ratio of 1:3 displayed a higher intensity of reflexes coming from anatase phase. This provides evidence that the anatase phase constituted a larger part of the material than the sample with the molar ratio of 1:1. Fe_3_O_4_/C/TiO_2_ (1:10) material was characterized by a lower intensity of reflexes coming from anatase phase, compared to other materials modified with titanium dioxide. The increase in the amount of TiO_2_ precursor to 10 mL did not increase the intensity of the anatase phase reflex.

Table 1 presents the percentage share of crystalline phases determined with Rietveld refinement. The maximum percentage of the anatase phase (78%) was observed in the sample with Fe_3_O_4_: TiO_2_ molar ratio of 1:3. The increase in the amount of TiO_2_ precursor did not affect the content of anatase crystalline phase in the sample. The decrease in crystalline anatase content down to 48% was observed in the sample with Fe_3_O_4_: TiO_2_ molar ratio of 1:10. This finding is consistent with earlier data described elsewhere [14].

Based on the half-width of reflections characteristic of both the magnetite phase and anatase, the average crystallite sizes of these phases were determined with the Scherrer equation and are shown in Table 1. The crystallite size of pure magnetite was approximately 15.6 nm. It was observed that the addition of carbon and titanium dioxide caused a decrease in average magnetite crystallite size from 15.6 to 9.2 nm. The average size of anatase crystallites was in the range of 11–15.2 nm.

BET analysis was used to determine the specific surface area of obtained materials and the results are shown in Table 2. Unmodified magnetite had the specific surface area of approximately 79 m^2^/g. The modification with carbon and titanium dioxide caused a significant increase in the specific surface area of obtained materials.

**Figure 2:**
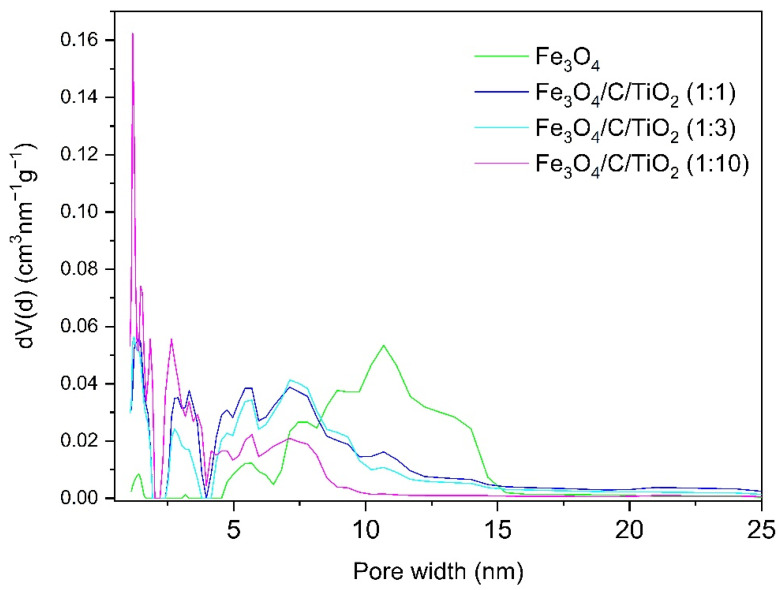
The pore size distribution calculated using the density functional theory (DFT) method [15].

Figure 2 shows the pore size distribution of pure magnetite (Fe_3_O_4_) and magnetite modified with titanium dioxide in different molar ratios (Fe_3_O_4_/C/TiO_2_). Nanocrystalline magnetite is a mesoporous material, with a pore size in the range of 5–15 nm [12]. The modification with titanium dioxide caused the pore size to decrease. The mesopores in modified materials had a size between 2 and 10 nm. As the amount of titanium dioxide increased, the share of mesopores decreased and that of micropores below 2 nm increased.

**Figure 3:**
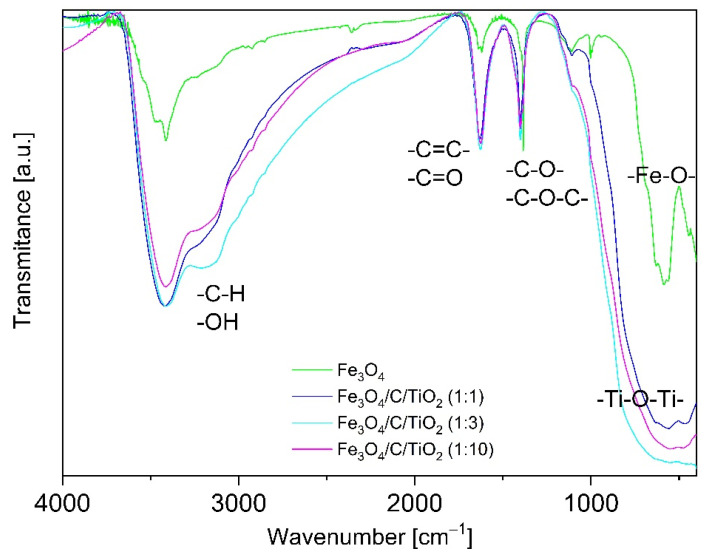
FT-IR spectra of pure magnetite and carbon and titanium dioxide modified magnetite in various molar ratios.

Figure 3 shows FT-IR spectra of magnetite covered with a carbon layer (Fe_3_O_4_) and magnetite modified with varied amounts of titanium dioxide. A broad band with a wavelength of approximately 3500 cm^−1^ was observed. This was ascribed to the stretching vibration corresponding to the presence of hydroxyl groups (OH) coming from water adsorbed on the surface [16,17].

A significant intensity increase in this band was observed in materials modified with carbon and titanium dioxide. This may have been due to the presence of CH groups, whose characteristic band is in the range of 2700–3300 cm^−1^ [17].

The FT-IR spectrum of unmodified magnetite had a characteristic reflex at the wavelength between 400 and 800 cm^−1^ coming from the bond of Fe–O. The spectra of materials containing titanium dioxide had a characteristic reflex in the range of 400–600 cm^−1^, which is typical of Ti–O–Ti bond stretching vibration. This band was relatively broader due to the overlapping of Ti–O peak along with the Fe–O peak mentioned above, proving the attachment of TiO_2_ on the surface of Fe_3_O_4_ [13].

The characteristic bands corresponding to the stretching vibration of double bonds of –C=C- and –C=O were observed in the range of 1500–1800 cm^−1^ [18]. In the range of 1000–1500 cm^−1^, there were stretching vibration bands coming from –C–O–C– and –C–O– single bonds, whose intensity increased in the materials modified with carbon and TiO_2_.

**Figure 4:**
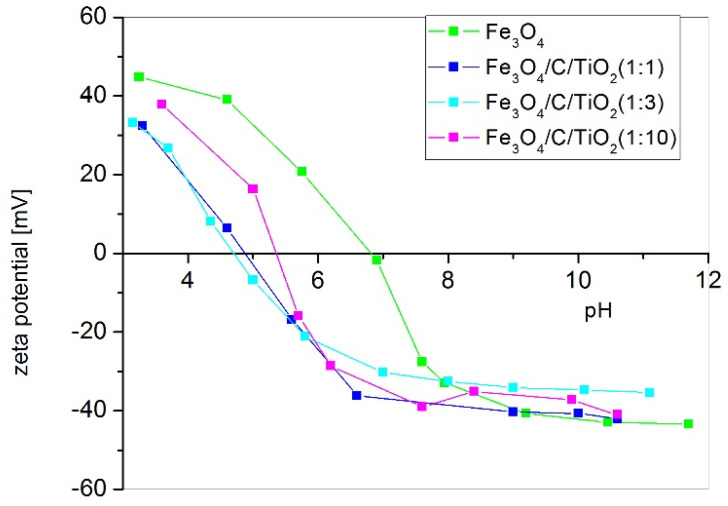
The zeta potential vs. pH value.

Figure 4 shows the typical correlations between the zeta potential and pH values obtained for magnetite and hybrid nanoparticles with titanium dioxide. The correlation between the zeta potential and pH can easily be seen. The isoelectric point (IEP) of magnetite is approximately at 6.81 pH and this finding is consistent with the literature [19]. When the surface of magnetite is covered with glucose and titanium dioxide, the isoelectric point is shifted towards lower pH, which is also in line with the literature data [20]. The increase in pH above the isoelectric point results in the surface of obtained nanoparticles having a negative potential. The farther the pH is from the isoelectric point, the greater the absolute value of the zeta potential is. The absolute zeta potential values affect nanoparticle dispersion in the solution. The higher the absolute zeta potential, the higher the dispersion of nanoparticles in the solution and the higher stability [20]. The nanoparticles of magnetite modified with glucose and titanium dioxide display a negative surface potential at pH = 7 with maximum absolute value. However, magnetite nanoparticles under these conditions have a negative charge and their absolute potential is very low.

**Figure 5:**
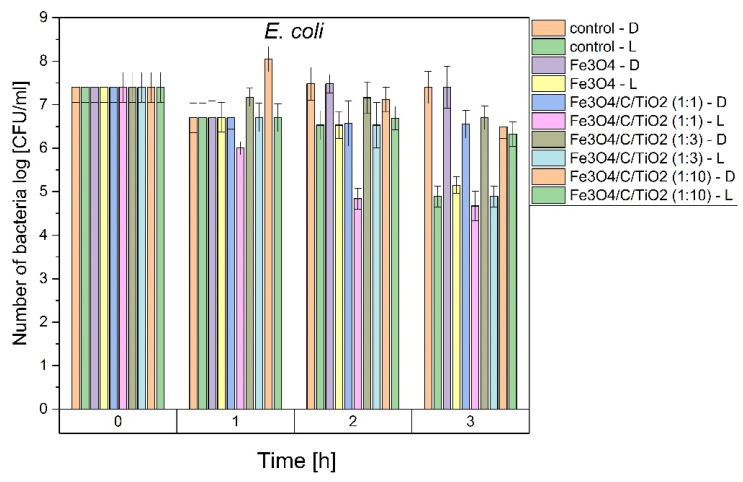

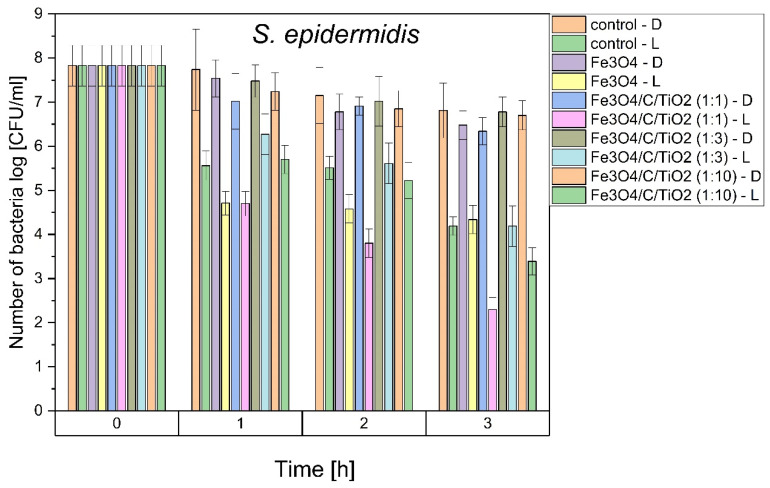
Antibacterial activity against *E. coli* and *S. epidermidis* of magnetite and glucose and titania modified magnetite, in dark and under UV-Vis irradiation solar light conditions.

In this study, *E. coli* and *S. epidermidis* were chosen as model bacteria to evaluate the photocatalytic antibacterial properties of the prepared Fe_3_O_4_/C/TiO_2_ under visible light irradiation. As shown in Figure 5, the survival rate of both studied bacteria suggested that the number of bacterial cells remained constant in the experiments conducted in dark conditions, indicating that there was no toxic effect to the bacterial cells for photocatalysts in the dark. In addition, it can be seen that UV-VIS light caused approx. 2 log reduction in bacteria number after 3 h. The photocatalytic performance of Fe_3_O_4_/C/TiO_2_ (1:1) compositions was improved but only against *S. epidermidis*. After 3 h, a 5 log reduction was observed (i.e., 99.999% of microbes were inactivated). This material is characterized by higher anatase contents and a small crystallite size. Our previous studies have shown that these parameters were the most decisive factors affecting the antibacterial activity of TiO_2_ photocatalyst used for water disinfection [21,22]. Higher activity against Gram-positive bacteria may be explained by the different structure of the cell wall and the lipid composition of bacterial cytoplasmic membranes (plasma membrane PM). It is well known that Gram-negative bacteria have an additional protective barrier of the outer membrane and their PM contains both anionic and zwitterionic phospholipids, while Gram-positive bacteria have only one membrane and PM contains predominantly anionic lipids [11,23]. For this reason, Gram-negative bacteria are more resistant to antimicrobial agents. Cationic particles (with a higher zeta potential) generally display more toxicity associated with increased permeability caused by the formation of phase boundary defects and membrane lipid rearrangements caused by the clustering of anionic lipids. It was proved (Figure 4) that the zeta potential of synthesized Fe_3_O_4_/C/TiO_2_ depends on pH. Hence, there is likely more than one factor contributing to the antibacterial action mechanism. As was shown by Belousov et al., bacteriostatic action induced by magnetite nanoparticles correlated with reaction environment (the used buffer). Magnetic nanoparticles changed the polarization structure of water inside microorganisms [24]. As shown in Figure 5, all tested materials presented better antibacterial properties against *E. coli* and *S. epidermidis* under UV-VIS irradiation than in dark conditions, which indicates photocatalytic effect. Many antibacterial studies were made using different Fe_3_O_4_ nanoparticles but results are inconclusive and depend on the method used. It was proved that they presented significant antibacterial activity against various types of microorganisms when they were applied in higher concentrations (from 10 to 15 mg/mL) than those used in the present study (0.1 mg /mL). However, only the agar diffusion method (ADM) or Kirby–Bauer test was applied [25,26,27]. This method is especially recommended for antibiotic or drug tests. It should be also noted that contact time between material and bacteria according to the standard test procedure is long (overnight incubation is favored). Based on these data, it can be concluded that total bacterial reduction in *E. coli* and *S. epidermidis* is possible also in dark conditions when contact time between bacterial species and Fe_3_O_4_/C/TiO_2_ is prolonged (24 h). Moreover, Ma et al. suggested that the presence of scavengers modulated the photocatalytic activity of Fe_3_O_4_-TiO_2_ nanosheets (TNS). Based on the obtained results they revealed that the major reactive species for disinfection under solar light were h^+^ and H_2_O_2_ which could oxidize the outer membrane of bacteria to a greater extent than •OH species [28]. They were also proved to exhibit more than 90% of removal efficiency toward *E. coli* after 120 min, at a concentration of photocatalyst of 0.1mg/mL. However, more importantly, the same efficiency was obtained even after five recycles. This high antibacterial performance resulted most likely from the used method and structure of obtained materials, due to the surface areas of Fe_3_O_4_-TNS and Fe_3_O_4_/C/TiO_2_ (1:1) obtained in this study being comparable, and amounted to 295.8 and 287 m^2^/g, respectively [28].

## 4. Conclusions

The results presented in this work are promising, showing that Fe_3_O_4_/C/TiO_2_ can be advantageous compared to a classical, plain photocatalyst and offers a powerful and challenging alternative for water disinfection under solar irradiation. It was proved that some Fe_3_O_4_/C/TiO_2_ composites possess good antimicrobial properties. However, the main advantage of magnetic nanomaterials that should be taken into account is the ease of separation from water after disinfection process even by application of magnetic field. On the one hand, it allows the release of chemicals (TiO_2_) to water to be avoided; on the other it, reduces operation cost by allowing their reuse. However, there are still open questions that should be answered to better understand the mechanisms of microbial inhibition and to test varied experimental conditions (e.g., optimum concentration, TiO_2_ content, and time of contact).

## Figures and Tables

**Table 1:** Average size of magnetite and anatase crystallites and their percentage share in the obtained materials.

Materials	Crystallite Size [nm]		Phase Composition [%]	
	Magnetite	Anatase	Magnetite	Anatase
Fe_3_O_4_	15.6	-	100	-
Fe_3_O_4_/C/TiO_2_ (1:1)	9.5	13.4	48	52
Fe_3_O_4_/C/TiO_2_ (1:3)	9.2	15.2	23	77
Fe_3_O_4_/C/TiO_2_ (1:10)	9.4	11.0	52	48

**Table 2:** Specific surface area and total pore volume of the obtained materials.

Materials	Specific Surface Area [m^2^/g]	Total Pore Area [cm^3^/g]
Fe_3_O_4_	79	0.302
Fe_3_O_4_/C/TiO_2_ (1:1)	287	0.431
Fe_3_O_4_/C/TiO_2_ (1:3)	197	0.321
Fe_3_O_4_/C/TiO_2_ (1:10)	282	0.260

## Data Availability

Not applicable.

## References

[1] The Sustainable Development Goal. https://unstats.un.org/sdgs/report/2021/goal-06/.

[2] Donde O.O., Atoni E., Muia A.W., Yillia P.T. (2021). COVID-19 pandemic: Water, sanitation and hygiene (WASH) as a critical control measure remains a major challenge in low-income countries. Water Res..

[3] Stankic S., Suman S., Haque F., Vidic J. (2016). Pure and multi metal oxide nanoparticles: Synthesis, antibacterial and cytotoxic properties. J. Nanobiotechnology.

[4] Gholami A., Mohammadi F., Ghasemi Y., Omidifar N., Ebrahiminezhad A. (2020). Antibacterial activity of SPIONs versus ferrous and ferric ions under aerobic and anaerobic conditions: A preliminary mechanism study. IET Nanobiotechnol..

[5] Khaloo S.S., Mozaffari S., Barekat A., Karimi F. (2015). Fabrication of a modified electrode based on multi-walled carbon nanotubes decorated with iron oxide nanoparticles for the determination of enrofloxacin. Micro Nano Lett..

[6] Mohan P., Mala R. (2019). Comparative antibacterial activity of magnetic iron oxide nanoparticles synthesized by biological and chemical methods against poultry feed pathogens. Mater. Res. Express.

[7] Huang K.S., Shieh D.B., Yeh C.S., Wu P.C., Cheng F.Y. (2014). Antimicrobial Applications of Water-Dispersible Magnetic Nanoparticles in Biomedicine. Curr. Med. Chem..

[8] Pang Y.L., Lim S., Ong H.C., Chong W.Y. (2016). Research progress on iron oxide-based magnetic materials: Synthesis techniques and photocatalytic applications. Ceram. Int..

[9] Liu Y. (2011). Magnetic-field induced formation of 1D Fe3O4/C/CdS coaxial nanochains as highly efficient and reusable photocatalysts for water treatment. J. Mater. Chem..

[10] Gnanasekaran L., Hemamalini R., Rajendran S., Qin J., Yola M.L., Atar N., Gracia F. (2019). Nanosized Fe_3_O_4_ incorporated on a TiO_2_ surface for the enhanced photocatalytic degradation of organic pollutants. J. Mol. Liq..

[11] Bokare A., Singh H., Pai M., Nair R., Sabharwal S., Athawale A.A. (2015). Hydrothermal synthesis of Ag@TiO_2_-Fe_3_O_4_ nanocomposites using sonochemically activated precursors: Magnetic, photocatalytic and antibacterial properties. Mater. Res. Express.

[12] Wojciechowska A., Lendzion-Bielun Z. (2020). Synthesis and characterization of magnetic nanomaterials with adsorptive properties of arsenic ions. Molecules.

[13] Sathya K., Saravanathamizhan R., Baskar G. (2017). Ultrasound assisted phytosynthesis of iron oxide nanoparticle. Ultrason. Sonochem..

[14] Lendzion-Bieluń Z., Wojciechowska A., Grzechulska-Damszel J., Narkiewicz U., Śniadecki Z., Idzikowski B. (2020). Effective processes of phenol degradation on Fe_3_O_4_–TiO_2_ nanostructured magnetic photocatalyst. J. Phys. Chem. Solids.

[15] Neimark A.V., Lin Y., Ravikovitch P.I., Thommes M. (2009). Quenched solid density functional theory and pore size analysis of micro-mesoporous carbons. Carbon.

[16] Wanag A., Rokicka P., Kusiak-Nejman E., Kapica-Kozar J., Wrobel R., Markowska-Szczupak A., Morawski A.W. (2018). Antibacterial properties of TiO_2_ modified with reduced graphene oxide. Ecotoxicol. Environ. Saf..

[17] Amézquita-Marroquín C.P., Torres-Lozada P., Giraldo L., Húmpola P.D., Rivero E., Poon P.S., Matos J., Moreno-Piraján J.C. (2020). Sustainable production of nanoporous carbons: Kinetics and equilibrium studies in the removal of atrazine. J. Colloid Interface Sci..

[18] Sharma G., Kumar A., Chauhan C., Okram A., Sharma S., Pathania D., Kalia S. (2017). Pecyin-crosslinked-guar gum/SPION nanocomposite hydrogel for adsorption m-cresol and o-chlorophenol. Sustain. Chem. Pharm..

[19] Liu J., Zhao Z., Jiang G. (2008). Coating Fe_3_O_4_ magnetic nanoparticles with humic acid for high efficient removal of heavy metals in water. Environ. Sci. Technol..

[20] Xu G., Zhang J., Li G., Song G. (2003). Effect of Complexation on the Zeta Potential of Titanium Dioxide Dispersions. J. Dispers. Sci. Technol..

[21] Markowska-Szczupak A., Rokicka P., Wang K., Endo M., Morawski A.W., Kowalska E. (2018). Photocatalytic water disinfection under solar irradiation by D-glucose-modified titania. Catalysts.

[22] Janus M., Markowska-Szczupak A., Kusiak-Nejman E., Morawski A.W. (2012). Disinfection of *E. coli* by carbon modified TiO_2_ photocatalysts. Environ. Prot. Eng..

[23] Epand R.M., Epand R.F. (2009). Lipid domains in bacterial membranes and the action of antimicrobial agents. Biochim. Biophys. Acta Biomembr..

[24] Belousov A., Voyda Y., Belousova E. (2016). Magnetite nanoparticles yield a significant bacteriostatic effect on microorganisms in relation to free radical lipid peroxidation. J. Transl. Sci..

[25] Taufiq A., Ikasari F.N., Yuliantika D., Sunaryono S., Mufti N., Susanto H., Suarsini E., Hidayat N., Fuad A., Hidayat A. (2019). Structural, Magnetic, Optical and Antibacterial Properties of Magnetite Ferrofluids with PEG-20000 Template. Mater. Today Proc..

[26] Asab G., Zereffa E.A., Abdo Seghne T. (2020). Synthesis of Silica-Coated Fe3O4 Nanoparticles by Microemulsion Method: Characterization and Evaluation of Antimicrobial Activity. Int. J. Biomater..

[27] Thukkaram M., Sitaram S., Kannaiyan S.K., Subbiahdoss G. (2014). Antibacterial Efficacy of Iron-Oxide Nanoparticles against Biofilms on Different Biomaterial Surfaces. Int. J. Biomater..

[28] Ma S., Zhan S., Jia Y., Zhou Q. (2015). Superior Antibacterial Activity of Fe_3_O_4_-TiO_2_ Nanosheets under Solar Light. ACS Appl. Mater. Interfaces.

